# Regulation of the Intranuclear Distribution of the Cockayne Syndrome Proteins

**DOI:** 10.1038/s41598-018-36027-6

**Published:** 2018-11-30

**Authors:** Teruaki Iyama, Mustafa N. Okur, Tyler Golato, Daniel R. McNeill, Huiming Lu, Royce Hamilton, Aishwarya Raja, Vilhelm A. Bohr, David M. Wilson

**Affiliations:** Laboratory of Molecular Gerontology, National Institute on Aging, Intramural Research Program, National Institutes of Health, 251 Bayview Blvd., Ste. 100, Baltimore, MD 21224 USA

## Abstract

Cockayne syndrome (CS) is an inherited disorder that involves photosensitivity, developmental defects, progressive degeneration and characteristics of premature aging. Evidence indicates primarily nuclear roles for the major CS proteins, CSA and CSB, specifically in DNA repair and RNA transcription. We reveal herein a complex regulation of CSB targeting that involves three major consensus signals: NLS1 (aa467-481), which directs nuclear and nucleolar localization in cooperation with NoLS1 (aa302-341), and NLS2 (aa1038-1055), which seemingly optimizes nuclear enrichment. CSB localization to the nucleolus was also found to be important for full UVC resistance. CSA, which does not contain any obvious targeting sequences, was adversely affected (i.e. presumably destabilized) by any form of truncation. No inter-coordination between the subnuclear localization of CSA and CSB was observed, implying that this aspect does not underlie the clinical features of CS. The E3 ubiquitin ligase binding partner of CSA, DDB1, played an important role in CSA stability (as well as DDB2), and facilitated CSA association with chromatin following UV irradiation; yet did not affect CSB chromatin binding. We also observed that initial recruitment of CSB to DNA interstrand crosslinks is similar in the nucleoplasm and nucleolus, although final accumulation is greater in the former. Whereas assembly of CSB at sites of DNA damage in the nucleolus was not affected by RNA polymerase I inhibition, stable retention at these sites of presumed repair was abrogated. Our studies reveal a multi-faceted regulation of the intranuclear dynamics of CSA and CSB that plays a role in mediating their cellular functions.

## Introduction

Cockayne syndrome (CS) is a rare, autosomal recessive disorder characterized by developmental impairment, multisystem progressive degeneration, and photosensitivity, without cancer predisposition^1^. There are two primary complementation groups, A and B, although there are other genetic defects that can give rise to CS-like clinical features (e.g. mutations in *XPB*, *XPD* and *XPG*, genes liked to the sun-sensitive and cancer-prone disorder, xeroderma pigmentosum (XP))^2,3^. The CSA protein (encoded by the *ERCC8* gene) is primarily composed of tryptophan-aspartic acid (WD) repeat motifs (seven in total), which are typically involved in facilitating the formation of multi-protein complexes^4^. Consistently, CSA is the substrate-targeting component of an E3 ubiquitin ligase complex, made up of DDB1 (DNA damage binding protein 1), RBX1 and CUL4A^5^. The CSB protein (encoded by the *ERCC6* gene) consists of a centrally-located ATPase-like domain, flanked by mostly non-conserved C- and N-terminal extensions. Biochemical assays have revealed that CSB possesses a DNA-dependent ATPase activity that is activated by a range of nucleic acid forms, including double-stranded DNA, duplex RNA and RNA/DNA hybrids, but not by single-stranded nucleic acid molecules^6^. While the precise molecular role of the ATPase activity of CSB remains unclear, complementation experiments have revealed its importance to CSB function, possibly in chromatin remodeling^7^ or lesion bypass^8^.

Based on identified protein interactions and certain cellular phenotypes, namely the profound sensitivity to ultraviolet C (UVC) radiation of CSA and CSB mutant cells, the CS proteins have been proposed to function at the interface of transcription and DNA repair, specifically in facilitating the removal of transcription-blocking lesions, such as ultraviolet (UV) photoproducts, in a process termed transcription-coupled nucleotide excision repair (TC-NER)^9^. In particular, CSA has been reported to interact with DDB1, as well as RNA polymerase II (RNAPII)^10,11^, while CSB has been shown to associate with RNAPII and the DNA repair and transcription factors, XPA, XPG and TFIIH^12–16^. Moreover, studies have indicated roles for CSB in gene regulation, namely in promoting RNA polymerase (I and II) elongation and chromatin remodeling^17–20^. The recent finding that CSA and CSB defects can lead to impaired transcription of G-rich templates has prompted a model whereby CS arises from inefficient transcription through G-quadraplex structures, which are found at particularly high frequency in ribosomal DNA (rDNA) in the nucleolus^21^. The stalled transcriptional events and the associated persistent DNA intermediates are then thought to lead to hyperactivation of poly(ADP-ribose) polymerase 1 (PARP1), consequent nicotinamide adenine dinucleotide (NAD^+^) depletion, and mitochondrial dysfunction, all of which participate in the etiology of CS^22^.

Consistent with their apparent biological roles, cellular localization studies have revealed that CSA exists primarily in the nucleus, with some presence in the nucleolus^23–27^. Experiments examining the distribution of CSB have similarly shown that this protein resides prominently in the nucleus, with possible nucleolar enrichment^26,28–31^. While mitochondrial localization and functions for both CSA and CSB have been demonstrated, their quantitative distribution to this organelle is low at best^32–34^, suggesting that the mitochondrial dysfunction associated with CS protein deficiency stems primarily from nuclear defects^22^. The aim of the current study was to identify elements of CSA and CSB that regulate the intracellular distribution of the proteins, as well as to determine whether CSB might respond uniquely to DNA damage within the nucleoplasm versus nucleolus.

## Results

### Features of CSB that Regulate Intracellular Distribution

**Figure 1:**
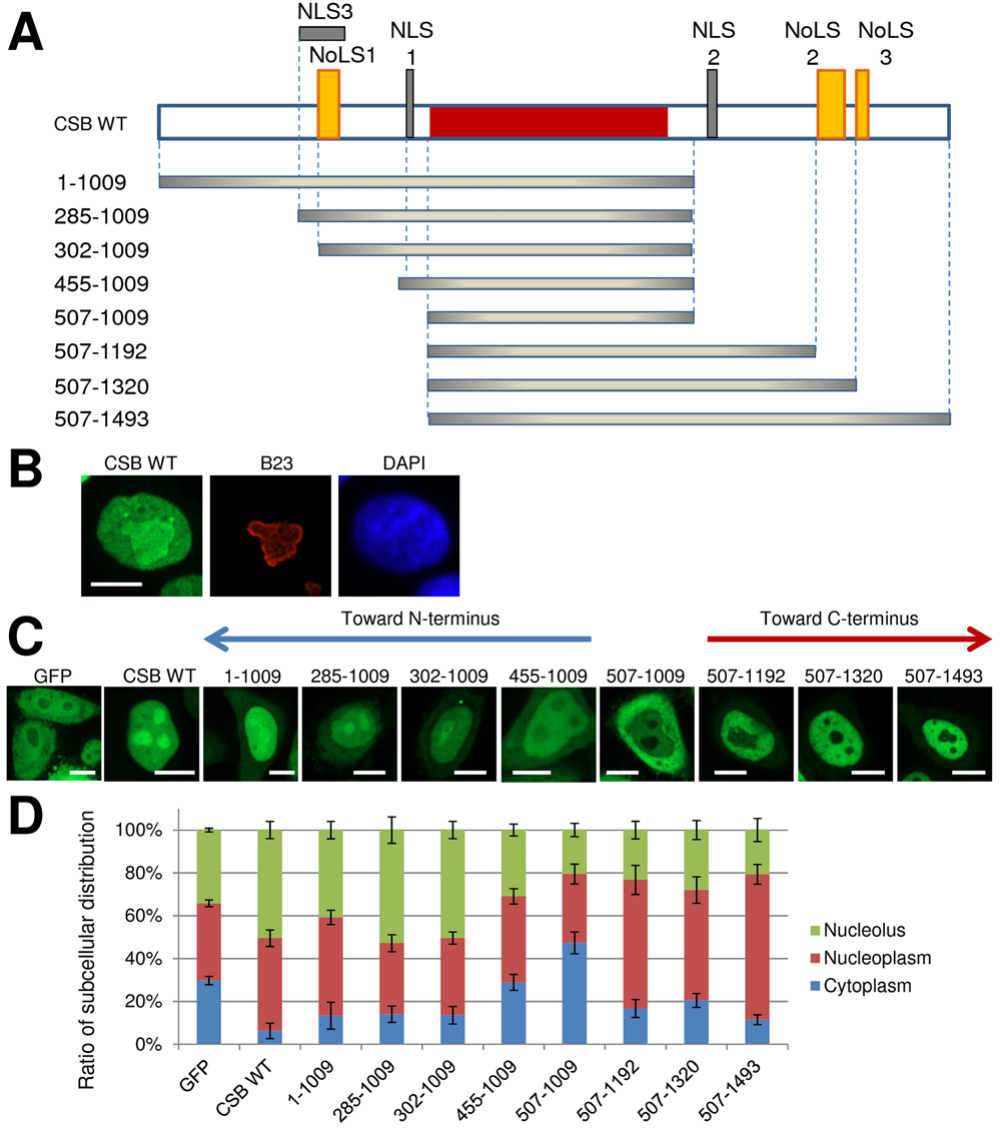
Regions of CSB predicted to contribute to intracellular localization. (**A**) Schematic of the domain structure of CSB WT (full-length CSB) and the CSB fragments used in this study. Eight CSB fragments, all of which include the ATPase domain (residues 507-1009, red), are shown. Regions of interest in CSB are: grey: NLS1: 467-481, NLS2:1038-1055, NLS3: 285-354; and orange: NoLS1: 302-341, NoLS2: 1189-1243, NoLS3: 1321-1343. (**B**) Nucleolar localization of CSB WT in HeLa cells. Cells were prepared following transfection of N-terminally GFP-tagged CSB WT and then visualized for GFP and stained with B23 (nucleophosmin) as a nucleolar localization marker or DAPI as a nuclear marker. Shown are representative images of GFP, B23 or DAPI in HeLa cells. Bar; 10 µm. (**C**) Localization pattern of CSB fragments in HeLa cells. Shown are representative images of GFP alone, GFP-tagged CSB WT or the eight N-terminally GFP-tagged CSB fragments in live cells. Bar; 10 µm. (**D**) Quantification of subcellular distribution profile of CSB fragments. The percentage of distribution of CSB fragments into nucleolus (green), nucleoplasm (red) or cytoplasm (blue) was determined from several live cell images (n = 37–70) from (**C**). Error bars indicate standard deviation (SD).

Past experimental studies have demonstrated nuclear localization for the CSB protein^26,28–31^, a property of CSB that is supported by computational prediction software, such as FUEL-mLoc^35^, Hum-mPLoc 3.0^35^, Iloc Animal^36^ and SCLpred^37^. To identify specific elements of CSB that might direct its intracellular distribution, we scanned its primary amino acid sequence using computational tools to uncover putative nuclear (cNLS Mapper^38^, NucPred^39^, SeqNLS^40^ and NLStradamus^41^) and nucleolar (NoD^42,43^) localization signals. In agreement with the initial report on CSB^44^, these programs identified two prominent nuclear localization signals, NLS1 and NLS2, as well as NLS3 and three nucleolar localization signals, NoLS1-3 (Fig. 1A). To determine the functionality of these consensus sequences, we employed basic truncation analysis, taking advantage of a previously described CSB-GFP (green fluorescent protein) fusion protein shown to localize to the nucleus, with nucleolar enrichment, and to complement the primary defects of CSB-deficient patient fibroblasts^28^. This nucleolar enrichment (see live cell images below) was confirmed by demonstrating co-localization of full-length CSB-GFP (CSB WT) with the nucleolar marker, B23 (nucleophosmin), using a standard immunostaining approach (Fig. 1B).

Using the ATPase domain as the starting point (residues 507-1009; Fig. 1A), this fragment was observed to exhibit a distribution pattern following transient transfection into HeLa cells that was similar to GFP alone (with nucleolar exclusion), yet an even greater presence in the cytoplasm (Fig. 1C,D), suggesting that the ATPase core in and of itself does not control intracellular localization. The addition of residues to the C-terminus of the ATPase domain that span NLS2 (507-1192) noticeably improved the nucleoplasm localization, while inclusion of NoLS2 (507-1320) and NoLS3 (507-1493) had little effect on the overall dispersal pattern in comparison to the 507-1192 fragment. These results suggest that NLS2 operates as a nuclear targeting signal, whereas the C-terminal NoLS have a limited role in directing CSB intracellularly, particularly to the nucleolus. Adding 52 amino acids to the N-terminus of the ATPase domain, which includes NLS1 (455-1009; Fig. 1A), improved slightly the nuclear localization in comparison to the 507-1009 ATPase fragment, although the 455-1009 fragment still displayed an overall distribution pattern similar to GFP alone (Fig. 1C,D). CSB fragments that harbored the overlapping NLS3 and NoLS1 elements in the N-terminal portion of the protein, but lacked the C-terminal residues (i.e., 1-1009 and 285-1009), acted most like CSB WT. Thus, while NLS1 appears to be a weak localization signal on its own, when combined with at minimum NoLS1 (the contribution of NLS3 appears to be minimal at best; compare 285-1009 to 302-1009), the protein is distributed in a manner most like CSB WT, including enrichment within the nucleolus, although perhaps slightly less nuclear due to the lack of C-terminal NLS2 (Fig. 1C,D). We note that prior work from our laboratory found that the C-terminal portion alone (residues 1010-1493) exhibited a localization pattern similar to CSB WT^45^. We observed here that the longer CSB fragment (507-1493), which contains the conserved ATPase domain, is mostly in the nucleoplasm, with nucleolar exclusion (Fig. 1). This finding suggests that in the absence of the ATPase core, NLS2 and presumably NoLS2 function efficiently at directing the C-terminal region (1010-1493) into the nucleus and nucleolus, respectively, consistent with data herein. It also implies that the enzymatic and/or DNA binding activities (or another unknown function) of the ATPase core modulate intranuclear distribution, a feature of CSB that requires further analysis going forward.

**Figure 2:**
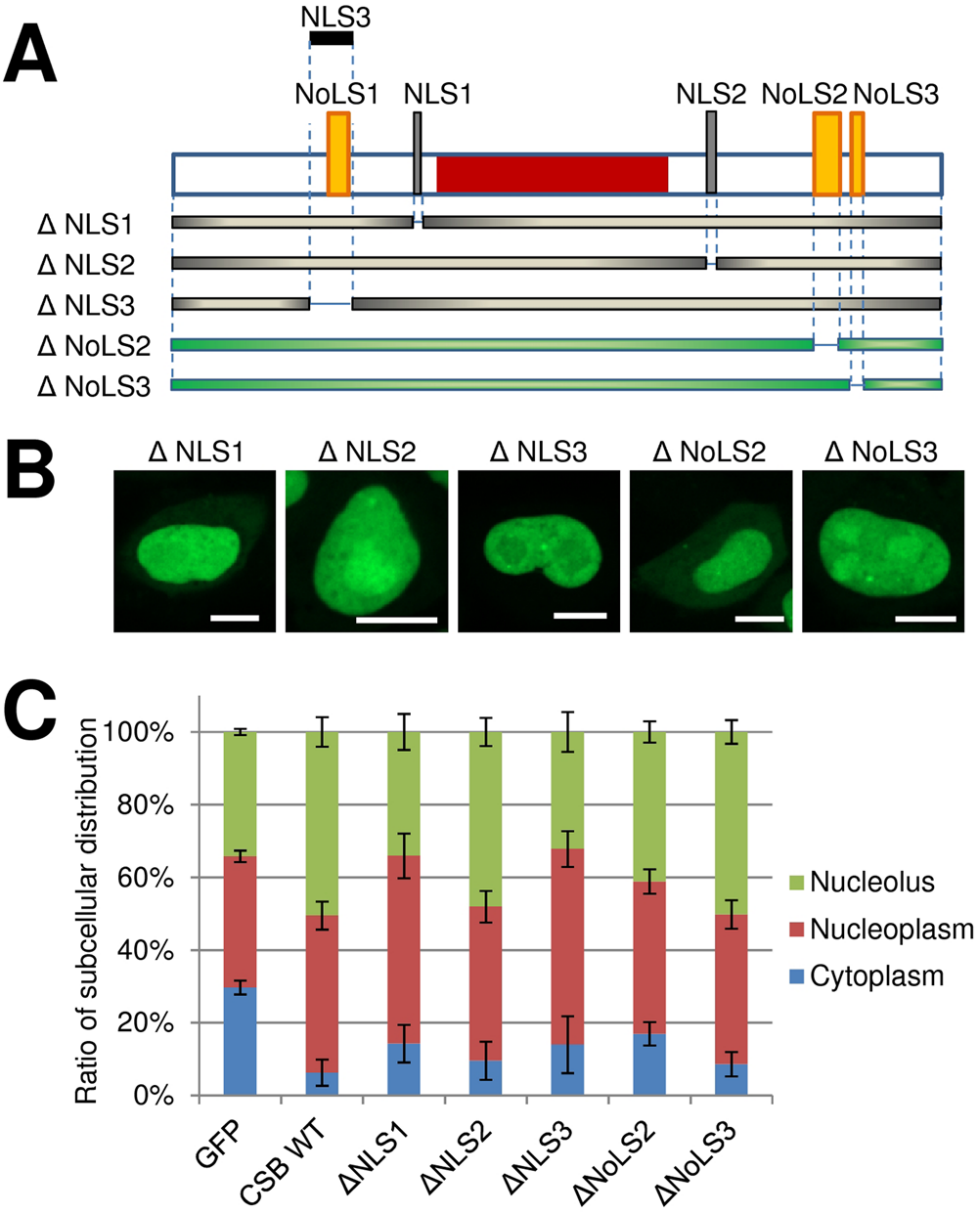
Contribution of consensus localization signals to CSB intracellular distribution. (**A**) Schematic of the domain structure of CSB, and N-terminally GFP-tagged CSB deletion fragments used in this study. See Fig. 1A for further description. (**B**) Localization pattern of CSB deletion mutants in HeLa cells. Shown are representative images of the five N-terminally GFP-tagged CSB mutants. Bar; 10 µm. (**C**) Quantification of subcellular localization profile of CSB fragments. The percentage of distribution of CSB fragments into nucleolus (green), nucleoplasm (red) or cytoplasm (blue) was analyzed from images (n = 51–77) of (**B**). Error bars indicate SD.

Performing a more selective deletion approach (Fig. 2A), we observed that removal of just NLS1 (residues 466-481) mainly resulted in a CSB variant that displayed reduced presence in the nucleolus (Fig. 2B,C), consistent with this element working in combination with NoLS1 to maximize nucleolar localization (see above). The NLS2 deletion (residues 1038-1055) had a minor effect on nucleoplasm presence (Fig. 2), consistent with this signal possibly operating to optimize nuclear targeting (see above). Removal of NLS3 (residues 285-354), which also removes NoLS1, resulted in a notable reduction in nucleolar presence of the CSB variant (Fig. 2), supporting a prominent role for NoLS1 in directing the protein to the nucleolus. Deletion of just NoLS2 or NoLS3 either reduced or had no effect on nucleolar distribution, respectively (Fig. 2), implying perhaps a minor role for the former signal in the context of the full-length protein.

**Figure 3:**
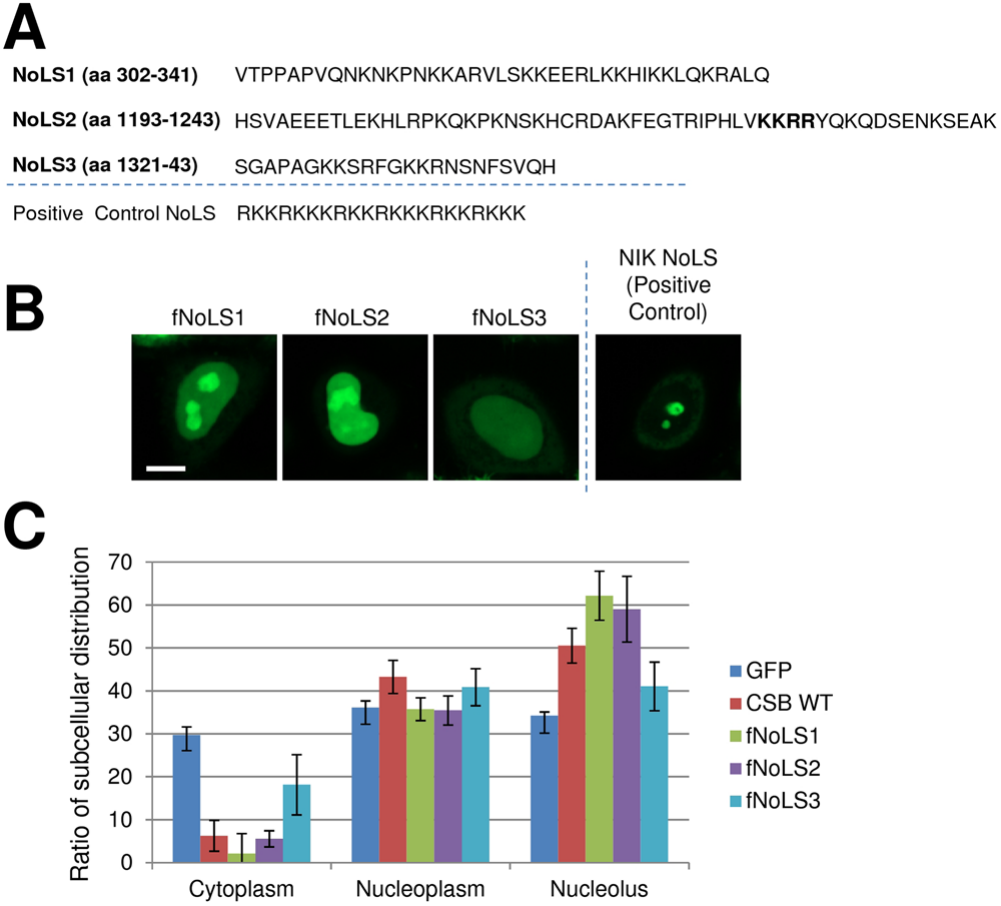
Targeting of the three CSB NoLS. (**A**) Schematic of the amino acid sequence of the three candidate NoLS in CSB and the NoLS sequence in NIK as a positive control. See Fig. 1A for further description. (**B**) Localization pattern of CSB NoLS-tagged GFP proteins in HeLa cells. Shown are representative images of the three CSB NoLS-tagged fragments and a typical NoLS targeting sequence. Bar; 10 µm. (**C**) Quantification profile of subcellular localization of CSB NoLS-tagged GFP proteins. The percentage of distribution of the different fusion proteins into the nucleolus (green), nucleoplasm (red) or cytoplasm (blue) was analyzed from images (n = 76–131) of (**B**). Error bars indicate SD. CSB WT is included as a comparison.

To more specifically address the role of the three candidate NoLS (Fig. 3A), we attached their respective sequences to GFP directly and subsequently determined the localization pattern of the fusion protein. Simultaneously, we checked whether GFP connected to a known NoLS would translocate into the nucleolus, specifically attaching the triple tandem repeats of the motif (RKKRKKK) from NF-κB inducing kinase (NIK) to GFP as a positive NoLS control^46^. As shown in Fig. 3B and quantified in 3C, the GFP fused to the NIK NoLS, or to either NoLS1 or NoLS2 from CSB, strongly localized to the nucleolus. The GFP-NoLS3 fusion, conversely, showed little nucleolar enrichment, although slightly higher than the GFP only control. Thus, in comparison to the GFP alone, our results indicate that both NoLS1 and NoLS2 are independent, strong nucleolar targeting signals, whereas NoLS3 is not, generally consistent with the studies presented above.

### Importance of Nucleolar Localization to CSB Function

**Figure 4:**
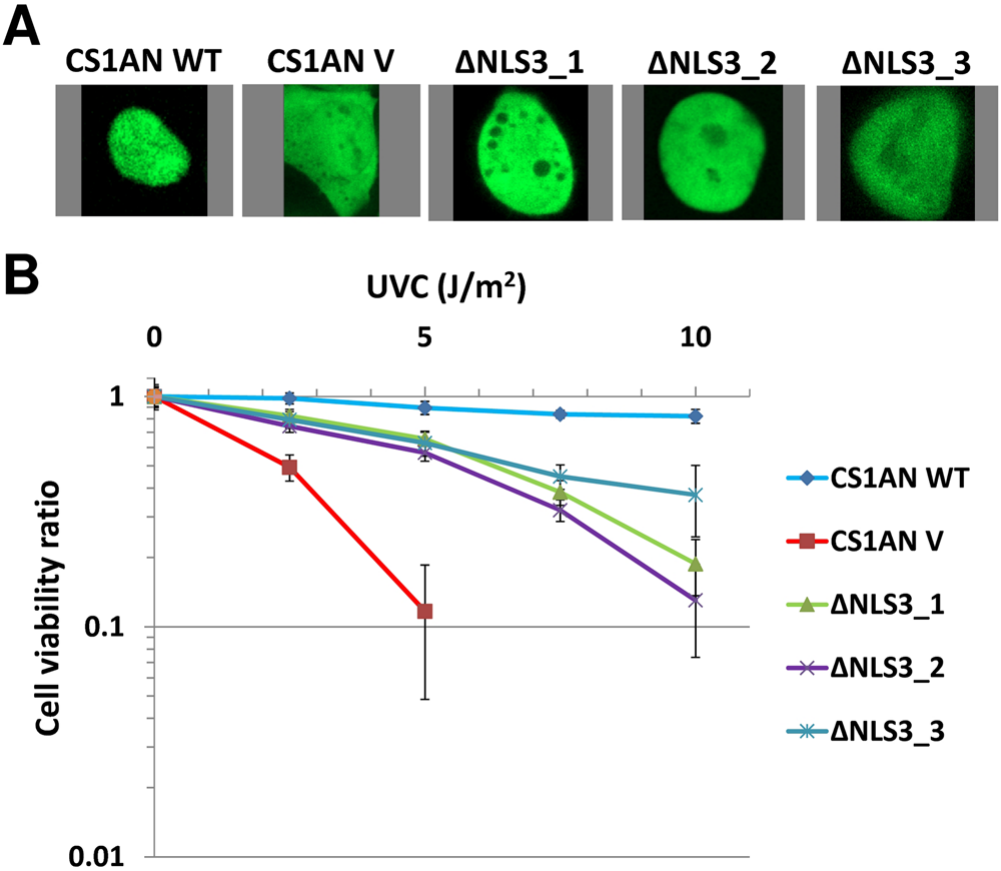
Nucleolar localization of CSB contributes to UVC resistance. (**A**) Representative confocal images of GFP distribution in CS1AN cells complemented with WT CSB, GFP vector (V) or CSB-ΔNLS3. The three independent CSB-ΔNLS3-complemented cells lines are designated 1, 2 and 3. (**B**) Viability of CS1AN cells complemented with WT CSB, GFP vector (V) or CSB-ΔNLS3 (1–3) following UVC irradiation. Cells were irradiated with the indicated dose of UVC and then assessed for viability using the CCK-8 assay (Dojindo Molecular Technologies, Inc., Rockville, MD). Each value plotted represents the average and standard deviation of 9 independent data points.

To determine the possible significance of the nucleolar localization of CSB, we established CSB-deficient CS1AN cells that stably express the CSB-ΔNLS3 GFP-tagged protein, which coincidentally lacks NoLS1 and exhibits reduced nucleolar localization (Figs 2A–C, 4A). While these cells grew similarly to both vector- and CSB WT-complemented CS1AN cells (unpublished observation), three independent CS1AN-CSB-ΔNLS3 cell lines displayed intermediate sensitivity to UVC irradiation (Fig. 4B). This result implies that nucleolar localization of CSB contributes to the UV resistance response.

### Recruitment of CSB to Sites of DNA Damage in the Nucleolus

Previously, using a laser microirradiation-confocal microscopy set-up, we characterized in detail the CSB protein response to a range of DNA lesions within the nucleoplasm^45^. Given the high degree of CSB in the nucleolus (Fig. 1B), we aimed to determine whether there was a distinct response of GFP-CSB to localized DNA damage in the nucleolus in comparison to the nucleoplasm. In light of the emerging role of CSB in DNA interstrand crosslink (ICL) repair and it’s robust response to this type of DNA damage^28^, we selected trioxsalen-induced ICLs (trioxsalen + UVA 354 nm laser) as our lesion of choice. For these studies, we employed a CSB-deficient patient cell line (CS1AN) that had been complemented with GFP-CSB WT and shown to exhibit normal CSB protein expression and UVC irradiation resistance^45^, consistent with the GFP-CSB WT fusion protein functioning normally.

**Figure 5:**
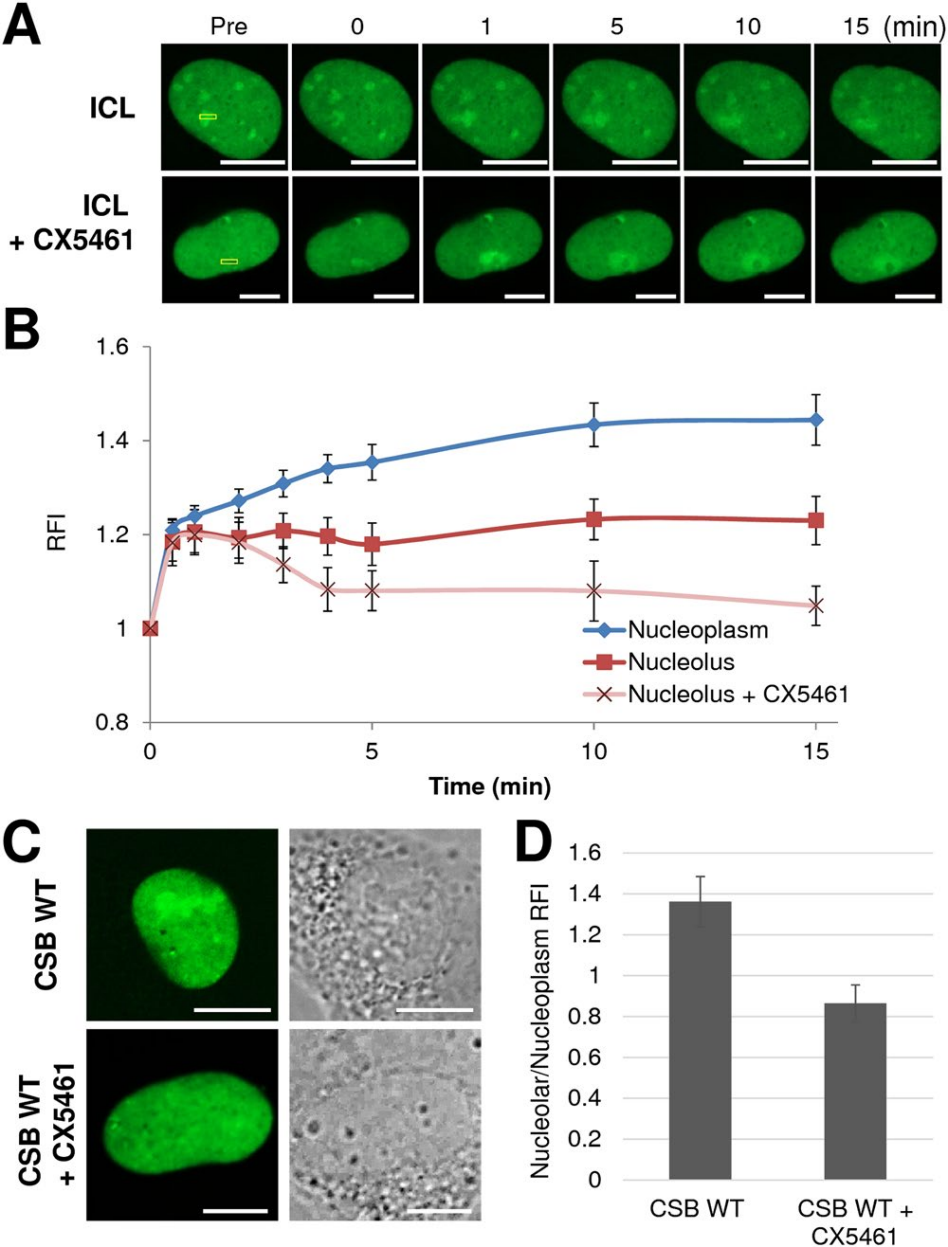
Response of CSB to ICLs in the nucleoplasm and nucleolus. (**A**) CSB recruitment and retention at ICLs (UVA laser + trioxsalen) with or without RNAPI inhibitor (CX5461) treatment. In CS1AN cells complemented with CSB WT, the indicated region (yellow box), spanning the nucleolus and nucleoplasm, was irradiated under the conditions specified. Shown are representative images of unirradiated cells (Pre), and the response of CSB to the DNA damage at 0, 1, 5, 10 and 15 min post-irradiation. Bar; 10 µm. (**B**) Quantification of CSB response to ICLs in the nucleolus and nucleoplasm. These graphs report the RFI of GFP-tagged CSB-GFP at the microirradiated area relative to unirradiated parts of either the nucleoplasm or nucleolus. Each data point is derived from at least 10 independent cells from two independent experiments. Error bars indicate SEM. (**C**) RNAPI inhibition reduces nucleolar distribution of CSB. Shown is a representative image of a CS1AN cell complemented with GFP-tagged CSB WT in the presence or absence of CX5461. Bar; 10 µm. (**D**) Quantification of the CSB intensity in the nucleolus versus nucleoplasm after CX5461 treatment. Thirteen independent cells were analyzed, and error bars indicate SEM.

To compare the nucleolar versus nucleoplasm CSB response, our approach involved microirradiating across the nucleolus (defined using phase contrast imaging and the stronger GFP-CSB signal) and into the neighboring nucleoplasm, so that an identical amount of UVA laser exposure could be applied simultaneously across both sub-nuclear compartments (Fig. 5A). In these experiments, we found that initial recruitment of GFP-CSB to sites of trioxsalen ICLs occurred in an identical kinetic profile up to 1 min post-irradiation in both the nucleoplasm and nucleolus (Fig. 5B). However, at this time-point, the nucleolar GFP-CSB signal had reached near-maximal intensity and was retained around this level for the duration of the time-course, whereas GFP-CSB continued to accumulate at sites of DNA ICLs in the nucleoplasm for ~10 min before plateauing, indicating a distinct and more robust overall response in this compartment.

Similar to RNAPII-directed TC-NER, we examined whether an active RNA polymerase I (RNAPI) is required for the CSB response to sites of DNA damage. Egly and colleagues have shown that CSB is in complex with RNAPI, TFIIH and XPG in the nucleolus to promote efficient ribosomal RNA (rRNA) production^26^. However, prior data has suggested that DNA damage within rDNA is poorly repaired in mammalian cells, and not via a TC-repair mechanism^47^. Using CX5461 to inhibit RNAPI activity, we found that the initial recruitment of GFP-CSB to ICLs was unaffected by polymerase inhibition up to 1 min post-irradiation in the nucleolus; however, CSB did not fully accumulate and was not retained at ICLs (Fig. 5B). In addition to affecting accumulation/retention of CSB at sites of trioxsalen-induced ICLs, RNAPI inhibition significantly diminished the nucleolar GFP-CSB signal, in some cases to levels below that observed in the nucleoplasm (Fig. 5C,D), although CX5461 treatment may adversely affect the nucleolar architecture as revealed by altered nucleolin (C23) staining (Fig. S1). Our studies indicate that active rDNA transcription is necessary for efficient CSB nucleolar localization, yet it does not appear to be essential for CSB recruitment to sites of DNA damage, only retention, consistent with perhaps the absence of a classic TC-repair mechanism in rDNA.

### Truncation of CSA Harms Protein Integrity

**Figure 6:**
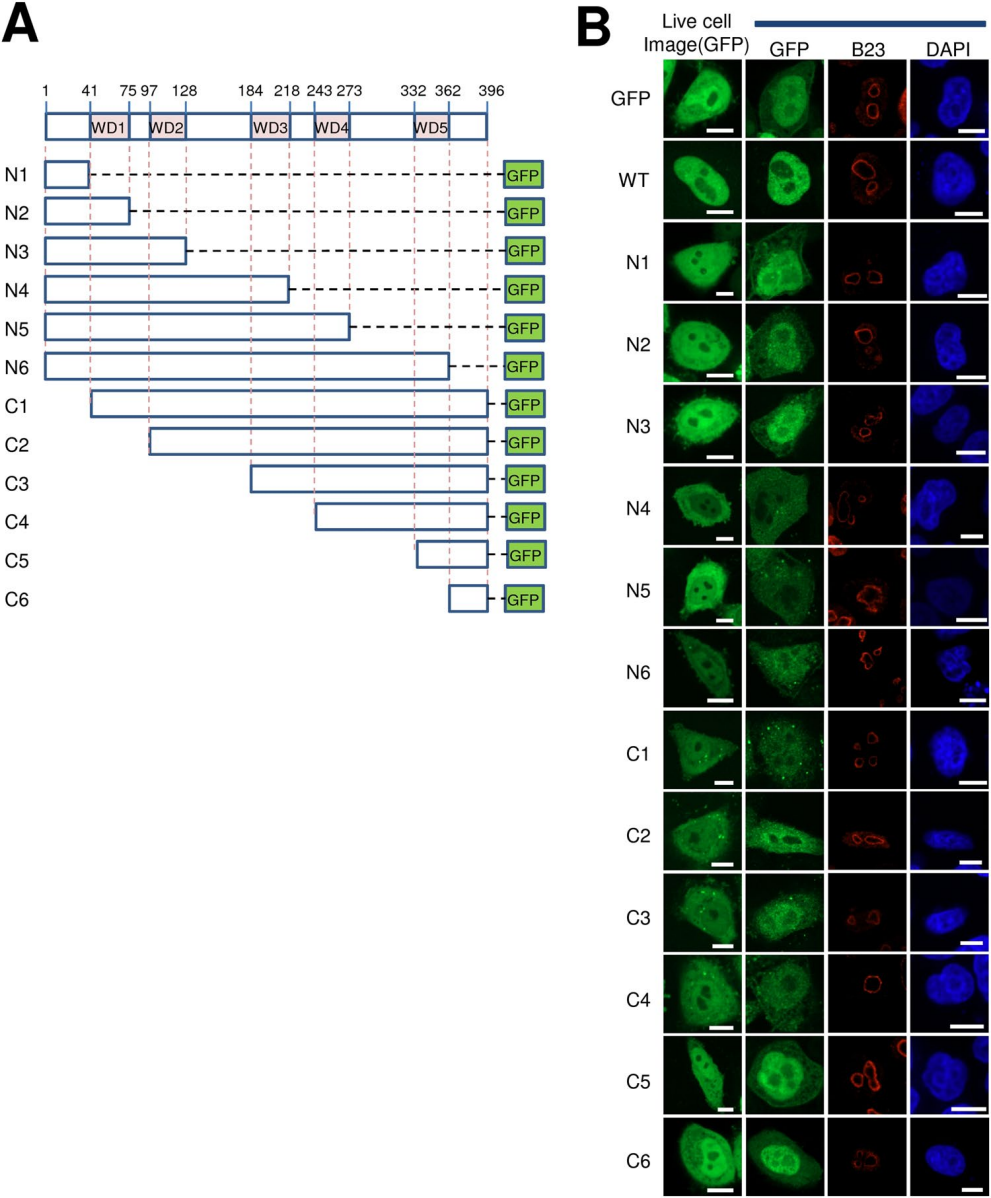
CSA protein is sensitive to deletion. (**A**) Schematic of the domain structure of CSA, and twelve C-terminally GFP-tagged CSA fragments (N1-6 and C1-6) used in this study. Regions of interest in CSA are the five WD40 motifs (41–75, 97–128, 184–218, 243–273, 332–362). (**B**) Localization pattern of CSA fragments. HeLa cells were transfected with the indicated plasmid, and then visualized for GFP distribution. Each panel includes a live cell image, as well as images of GFP, B23 and DAPI. Bar; 10 µm.

Work from our lab, as well as others, has indicated that full-length CSA is primarily localized in the nucleus^27,45,48^. However, using the computational methods noted earlier to identify consensus targeting sequences, we were unable to uncover any putative NLS within the CSA protein. Furthermore, subcellular localization prediction programs provide a mixed picture on the likely distribution of CSA, with FUEL-mLoc, Hum-mPLoc 3.0 and SCLpred predicting nuclear localization, and Iloc Animal predicting cytoplasmic or cytoskeletal. To experimentally determine whether there is a region within CSA that mitigates its nuclear distribution, we created a series of truncation mutants (shown in Fig. 6A) using a previously-described CSA-GFP system proven to be functional^45^. These studies found that any form of fragmentation of CSA leads to abnormal intracellular distribution, with a high degree of cytoplasmic localization similar to what is seen with GFP alone (Fig. 6B). These results imply that CSA consists of a tight globular protein conformation that is intolerant of any significant deletion, supporting the inference that nonsense mutations observed in the *ERCC8* gene of CS patients are non-functional^1,49^.

### DDB1 Stabilizes and Directs CSA to Chromatin in Human Cells

**Figure 7:**
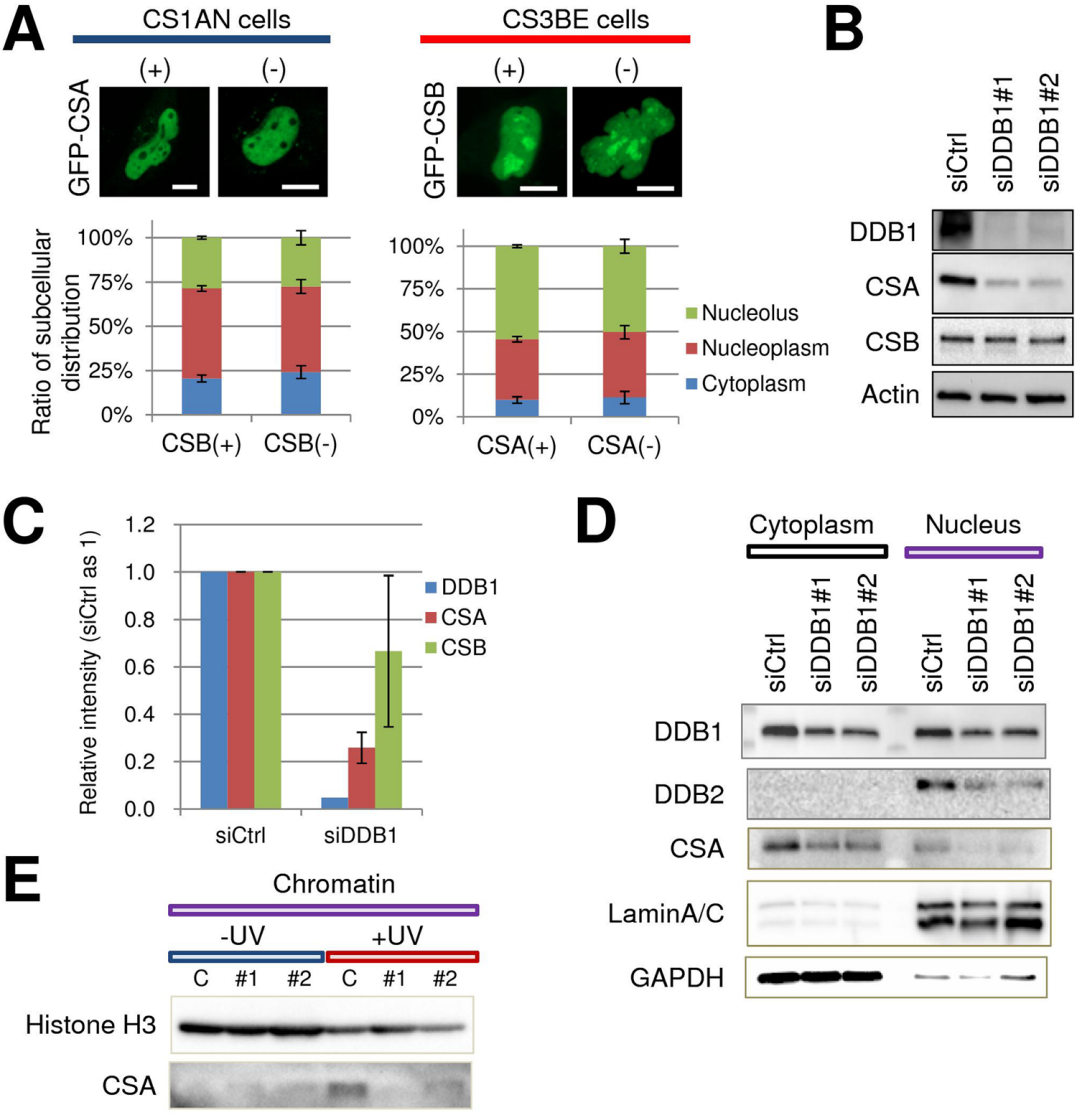
CSA is dependent on DDB1 for stability and chromatin association after UV irradiation. (**A**) The intracellular localization of CSA or CSB is not influenced by the other. (Left) Images and quantification of subcellular distribution profile of GFP-tagged CSA expressed in CSB-deficient or CSB-complemented CS1AN cells. (Right) GFP-tagged CSB expressed in CSA-deficient or CSA-complemented CS3BE cells. The percentage distribution of CSA (left) or CSB (right) in the nucleolus (green), nucleoplasm (red) or cytoplasm (blue) was determined from several live cell images (n = 21–27). Error bars indicate SD. (**B**) DDB1 deficiency leads to reduced CSA protein levels. Expression of CS proteins as determined by western blot analysis of whole cell extracts prepared from U2OS cells following treatment with control siRNA (siCtrl) or DDB1 siRNA (#1 or #2). (**C**) The relative ratio of DDB1, CSA or CSB in siDDB1-treated cells relative to the control siRNA line (set as 1) is shown. Error bars indicate SD (n = 4, made up of 2 independent runs each of siDDB1 #1 and #2). (**D**) Both CSA and DDB2 are reduced following knockdown of DDB1. After transfecting either the siRNA control (siCtrl) or siDDB1 (#1 or #2), U2OS cells were fractionated into cytoplasmic and nuclear fractions. Western blotting was then conducted for the indicated protein, with LaminA/C indicating nuclear enrichment and GAPDH verifying cytoplasm enrichment. (**E**) CSA chromatin association following UV treatment is impaired following DDB1 knockdown. Western blot analysis of chromatin fractions from U2OS cells with or without UV exposure (15 J/m^2^) was performed against histone H3 (chromatin marker) and CSA. C = siCtrl, #1 = DDB1 siRNA#1, #2 = DDB1 siRNA#2.

Since some reports suggest that CSA and CSB exist in the same complex in cells^11,15^, we speculated that the CS proteins might regulate one another’s localization. To examine this hypothesis, we compared the intracellular distribution of a GFP-tagged CS protein in the opposing CS-deficient or -complemented cell line. Specifically, we observed that the CSA-GFP distribution pattern was similar when expressed in either a CSB-deficient or CSB-complemented CS1AN patient cell line (Fig. 7A, left). Likewise, the intracellular localization of GFP-CSB did not differ in either CSA-deficient or CSA-complemented CS3BE fibroblast lines (Fig. 7A, right). These results indicate that CSA and CSB do not influence the localization pattern of one another.

Since it appears unlikely that CSA harbors a consensus targeting sequence (see above), we determined whether its binding partner DDB1 might direct CSA to the nucleus. DDB1 (aka p127) participates in DNA repair, either in complex with DDB2 (p48) or as part of the E3 ubiquitin-protein ligase complex that includes CUL4A-RBX1^5^. In the case of the former, the DDB1-DDB2 heterodimeric DNA damage-binding complex participates in global genome NER, where defects in its functionality can give rise to XP complementation group E. When DDB1-CUL4A-RBX1 associate with CSA, the complex is believed to be targeted to sites of ongoing transcription-associated DNA repair. To examine whether DDB1 might participate in regulating CSA localization, we employed two different siRNA sequences to knockdown DDB1 in U2OS cells (Fig. 7B). Strikingly, we found that DDB1-deficiency (~95% down) resulted in a consistent and significant decrease (~75%) in CSA protein levels, with only a potentially minor decrease in CSB (Fig. 7C). To examine CSA intracellular distribution, we fractionated DDB1 knockdown or control cells into their cytoplasmic and nuclear compartments. These studies confirmed a coincident DDB1 and CSA reduction, though the relative distribution pattern of CSA remained similar (Fig. 7D), suggesting that DDB1 did not directly modulate CSA intracellular localization. We note that DDB1 knockdown also resulted in lower DDB2 levels (Fig. 7D).

For a more detailed look at CSA distribution in situations of DDB1 deficiency, we prepared chromatin fractions from DDB1 knockdown cells or control cells with or without UV treatment. In the absence of UV, neither CSA nor CSB accumulated in the prepared chromatin fraction (Fig. S1). Following UV irradiation, both CSA and CSB accumulated in the chromatin fraction in siRNA control cells. However, in the DDB1-deficient cells, CSA protein association with chromatin was markedly reduced (>90% down in comparison to the control cells; Fig. 7E), even when taking into account the already lower CSA protein levels, whereas CSB bound to chromatin with similar avidity independent of DDB1 status (Fig. S1). This finding suggests that DDB1 determines both the stability and DNA damage-induced chromatin association of CSA, but not CSB.

## Discussion

Prior work reported that both CSA and CSB localize to the nucleus, with the former predominantly in the nucleoplasm and the latter possibly enriched in the nucleolus^23–27^. These findings are consistent with the CS proteins operating primarily in DNA repair and RNA transcription, perhaps more so for rDNA^21^. However, little has been done previously to define the molecular elements that dictate the observed distribution patterns of CSA and CSB. Computational analysis of CSB identified a set of putative NLS and NoLS consensus targeting sequences. Our complementary approaches, which included traditional deletion/truncation methods, revealed that both NLS1 and NLS2 contribute to CSB nuclear localization, and that NoLS1 is the major nucleolar targeting element, likely in cooperation with NLS1. Complementation studies in CS1AN CSB mutant cells revealed that this nucleolar localization is important in the resistance response to UVC irradiation. As for CSA, while three of four computational tools indicated nuclear localization, neither computational methods nor truncation analysis uncovered an obvious NLS or NoLS in CSA; instead, it was found that the protein was exquisitely sensitive to any form of deletion, consistent with a highly-organized protein structure^5^.

Multiple groups have reported that CSA and CSB can localize in mitochondria, particularly in response to oxidative stress^32–34^. We have not explored this aspect exhaustively using our system, but saw no obvious or increased targeting of the any of the GFP fusions (either WT or the truncations/deletions) to this organelle, implying, at best, a low presence of CSA or CSB in mitochondria during unstressed conditions, and perhaps the lack of a true mitochondrial targeting sequence. Indeed, the current data favor a model in which defects in the resolution of transcription-blocking structures (e.g. DNA damage or G-quadraplexes) within the nuclear genome lead to PARP1 hyper-activation and consequent mitochondrial dysfunction, cellular events that likely underlie the clinical pathologies associated with CS and possibly other genetic disorders^21,22^. Clearly, future studies need to identify the mitochondrial targeting mechanisms for CSA and CSB to firmly establish a function for these two proteins in this intracellular compartment.

Since no obvious consensus localization signals were found in CSA, we explored the possibility that certain binding partners direct CSA to the nucleoplasm. Our studies found that knockdown of DDB1, which interacts with CSA as part of the CUL4A-RBX1 E3 ubiquitin-protein ligase complex in TC-NER^5^, results in a pronounced and concomitant reduction in both the CSA and DDB2 protein levels. In addition, DDB1 deficiency was found to reduce chromatin association of CSA following UV irradiation, implying that DDB1 plays a critical role in both maintaining CSA protein stability and organizing CSA at sites of DNA damage. This finding would suggest that mutations in *DDB1* could give rise to phenotypes resembling aspects of both XP and CS, although to our knowledge pathogenic mutations have not yet been identified in this gene.

Prior experiments by the Vermeulen lab^50^ demonstrated a transcription-dependent enrichment of GFP-CSB at locally-induced oxidative DNA damage in both the nucleoplasm and nucleolus. We investigated the response of CSB in these two compartments to sites of ICLs, a DNA lesion likely to elicit a transcription-associated repair process^28,29,45^. Although initial recruitment of CSB to ICLs is kinetically identical in both compartments, CSB recruitment to ICLs in the nucleolus stabilized rapidly, plateauing ~1 min post-irradiation, whereas CSB continued to accumulate up to ~10 min post-irradiation at localized DNA damage in the nucleoplasm (Fig. 5A,B). These distinct response patterns may be related to the chromatin nature of the genome within the nucleolus and nucleoplasm. In particular, the bulk of the nucleolus contains actively transcribed rDNA arrays, which mandate an open chromatin state for robust RNAPI transcription, and thus potentially has a lesser need for CSB as a chromatin remodeler to facilitate repair. Conversely, the nucleoplasm contains a greater diversity of chromatin states, and therefore may require a higher concentration of CSB to enable an efficient repair response.

Prior work using α-amanitin, an RNAPII inhibitor, demonstrated a RNAPII-dependent recruitment of CSB in the nucleoplasm to sites of complex lesions, such as UV-induced damages^51^ and DNA ICLs^28,45^. However, in the studies here, we found that initial recruitment of CSB to sites of ICLs in the nucleolus was not abrogated by inhibition of RNAPI via CX5461, although total CSB accumulation and retention at ICLs were suppressed by RNAPI inhibition. This pattern of assembly and retention at ICLs in the nucleolus is similar to what we had seen previously for CSB at sites of oxidative DNA damage in the nucleoplasm upon inhibition of RNAPII, with initial recruitment seemingly unaffected, followed by a suppression of accumulation and retention at the damage site. Since initial recruitment and assembly are largely unchanged, our data support the previous conclusion that rDNA is not repaired in a classic transcription-coupled response^47^. Nevertheless, it appears that RNAPI activity is necessary for efficient localization of CSB to the nucleolus, possibly due to its role in facilitating rDNA transcription or maintaining nucleolar architecture, and at retaining CSB at sites of ongoing rDNA repair.

In closing, our studies have revealed mechanisms that regulate the intranuclear localization or response of CSA and CSB. Even though these proteins are presumed to operate in a common molecular pathway, since they give rise to virtually identical clinical phenotypes when defective, our data indicate that neither of them regulates the distribution pattern of the other, implying unique and specific mechanisms for intracellular trafficking: CSA (binding partners) and CSB (consensus localization sequences). Thus, in addition to the complex mechanisms that regulate protein recruitment to sites of blocked transcription, such as during TC-NER, there are equally complex systems that facilitate distribution of CSA and CSB within the cell. Whether altered intracellular localization of pathogenic variants in either CSA or CSB contributes to the molecular defects that give rise to CS awaits further investigation.

## Materials and Methods

### Plasmid constructs

Creation of N-terminally GFP-tagged CSB 1-1009 or CSB 455-1009 plasmids was previously reported^45^. To create other truncation mutants of CSB, the appropriate coding region was amplified from pCSB-GFP (CSB WT)^28^ using PrimeSTAR Max DNA polymerase (TaKaRa, Shiga, Japan) and the primer sets described in Table S1. PCR products were digested and subcloned into the XhoI and BamHI restriction sites of pEGFP with linker 1^28^. The ligation reaction was transformed into NEB 5-alpha High Efficiency Competent *E. coli* (competent cells, New England Biolabs (NEB), Ipswich, MA), and the nucleotide sequences of independently isolated plasmids were confirmed at Eurofins Genomics (Huntsville, AL). To create the deletion mutants for the NLS or NoLS in CSB (pCSB-ΔNLS1, pCSB-ΔNLS2, pCSB-ΔNLS3, pCSB-ΔNoLS2 or pCSB-ΔNoLS3), an inverse PCR method was employed, where the DNA harboring a specific deletion in the *CSB* coding region was amplified from pCSB-GFP by the polymerase above and a corresponding primer set (Table S1). The PCR products were digested with NotI and DpnI, and ligated accordingly. To create the plasmids with potential NoLS fragments (pGFP-fNoLS1, pGFP-fNoLS2 or pGFP-fNoLS3), which express N-terminal-tagged GFP proteins, appropriate NoLS oligonucleotide sets (Table S1) were annealed, and the resulting DNA fragments were ligated into the XhoI and BamHI restriction sites of pEGFP with linker 1. The ligated DNA was transformed and sequence confirmed as above.

To create C-terminal GFP-tagged expression plasmids of CSA truncated mutants (CSA N2 to N6 or C1 to C5), the *CSA* coding region was amplified from pCSA-GFP^45^ as above using the corresponding primer sets (Table S1). The PCR products were digested accordingly and subcloned into the restriction sites of pAcGFP (Clontech, Mountain View, CA). To create the remainder of the constructs (CSA N1 or C6), the oligonucleotide set of CSA N1 or C6 was annealed, and the fragments were then ligated into the specific restriction sites of pAcGFP. The ligated DNA was transformed and sequenced confirmed as above.

### Cell lines and maintenance

HeLa and U2OS were grown in normal culture media: high glucose Dulbecco’s Modified Eagle Medium (DMEM) with 10% fetal bovine serum (FBS) and 1% penicillin/streptomycin. The SV40-transformed CSA-deficient CS3BE cell lines with either relevant vector or the untagged CSA protein, and the SV40-transformed CSB-deficient CS1AN cell lines with either relevant vector or non-tagged or GFP-tagged CSB protein have been described previously^34,45,52^. These cell lines were grown in normal culture media with 100 μg/mL geneticin. The three CSB-ΔNLS3 CS1AN complemented cell lines were created by transfecting pCSB-ΔNLS3 into SV40-transformed CS1AN cells using the JetPrime reagent (Polyplus-transfection, Illkirch, France). After 48 hr of incubation at 37 °C without drug, transfected cells were re-plated on a 15 cm plate containing 10% FBS media with geneticin (800 µg/ml) for selection, and Individual clones were isolated and expanded using standard techniques. All cell lines were grown in a cell culture incubator maintained at 5% CO_2_ and 37 °C.

### Transfection and localization analysis

To determine GFP-tagged CSB localization patterns, HeLa cells were precultured in a 35 mm glass-bottom dish (MatTek, Ashland, MA), transfected with the indicated plasmid using the JetPrime reagent (see above), and cultured an additional day before visualizing with confocal microscopy^45^. All images were photographically recorded and analyzed using the Volocity software 6.3 (PerkinElmer, Waltham, MA) or Fiji^53^.

### Laser microirradiation and confocal imaging

The platform for the recruitment/retention studies using laser microirradiation and confocal imaging has been described previously^45^. CS1AN cells complemented with CSB-GFP were grown in the presence of 6 μM trioxsalen for 1 hour and/or 1 μM CX5461 for 24 hr prior to microirradiation. Data were analyzed and reported as relative fluorescence intensity (RFI) of the fluorescent-tagged protein at the microirradiated area relative to unirradiated (background) parts of the nucleus, with the specific cell numbers being provided in the relevant figure legend.

### DDB1 knockdown and chromatin fractionation

Two DDB1 siRNA oligonucleotides (siDDB1-1, 5′-UAACAUGAGAACUCUUGUC-3′; siDDB1-2, 5′-AUAAACAGCAGGUCCUUGC-3′) were supplied by Dharmacon (Lafayette, CO)^54^. The indicated DDB1 siRNA or the non-target control siRNA #1 (Dharmacon) was transfected into U2OS with INTERFERin (PolyPlus-Transfection) as described^55^. Briefly, 0.1 nmol siRNA was diluted in 400 µL Opi-MEM serum reduced media (Thermo Fisher Scientific Inc.), and then mixed with 20 µL INTERFERin transfection reagent. After a 15 min incubation at room temperature, the siRNA/INTERFERin solution was added to the cells for three days in 4 mL of DMEM medium with 10% FBS and 1% penicillin/streptomycin. To create whole cell extracts, cells were washed with cold PBS twice, and lysed in 1X RIPA buffer (Cell Signaling Technology, Danvers, MA). To prepare cytoplasmic and nuclear fractions, cells were processed using the NE-PER Nuclear and Cytoplasmic Extraction Kit (Cat# 78833) as specified by the manufacturer (Thermo Fisher Scientific Inc.). In the chromatin experiments, U2OS cells were irradiated with 15 J/m^2^ UVC (15 sec of 1 J/m^2^/sec) and harvested 5 hr post-irradiation, and the chromatin fraction was then prepared using a commercial fractionation kit (Cat# 78840, Thermo Fisher Scientific Inc.). The concentration of the different fractions was determined using the Bio-Rad protein assay kit (Bio-Rad Laboratories, Hercules, CA). Thirty µg total protein was typically separated on a 4–15% SDS-PAGE gel (Bio-Rad Laboratories) and transferred to a PVDF membrane for western blotting.

### Immunostaining and western blotting

The immunostaining and western blotting procedures were described previously^28,45^, and the relevant primary antibodies are provided in Table S2.

## Electronic supplementary material

Supplementary Material
