# Supplementary material for: Regulation of the Intranuclear Distribution of the Cockayne Syndrome Proteins
Source: Sci Rep. 2018 Nov 30;8:17490. doi: 10.1038/s41598-018-36027-6 (PMC6269539; doi:10.1038/s41598-018-36027-6)
Supplement: Supplementary file 1 — Supplementary Material [file 41598_2018_36027_MOESM1_ESM.pdf]

Regulation of the Intranuclear Distribution of the Cockayne Syndrome Proteins

Teruaki Iyama, Mustafa N. Okur, Tyler Golato, Daniel R. McNeill, Huiming Lu, Royce Hamilton, Aishwarya Raja, Vilhelm A. Bohr, David M Wilson III

Laboratory of Molecular Gerontology, National Institute on Aging, Intramural Research Program, National Institutes of Health, Baltimore, MD 21224 USA

Table S1. Oligonucleotide list for CSA and CSB GFP-tagged expression systems. Relevant plasmid names are listed, and can be matched to identify PCR primer pairs. Subcloning (restriction) site is indicated. All oligonucleotides were purchased from Integrated DNA Technologies (Coraville, IA).

Oligo name	Nucleotide Sequence	Plasmid name	Restriction site
CSA Fw	ATTTTGCTAGCCGCCATGCTGGGGTTTTTGTCCGCACGC	CSA N2-6	NheI
CSA N2 Rv	TTGAACAAGCTTGTCATAAAGTACAATCAC	CSA N2	HindIII
CSA N3 Rv	TTGAACAAGCTTATCCCATACTTTCAGAG	CSA N3	
CSA N4 Rv	TTGAACAAGCTTTCTTCTCACATCCCATAATT	CSA N4	
CSA N5 Rv	TTGAACAAGCTTATTCCAGAGCCTCATTCGATTATC	CSA N5	
CSA N6 Rv	TTGAACAAGCTTAACCCAAGCCAGAATGTTGCAG	CSA N6	
CSA C1 Fw	ATTTTCTCGAGGCCACCATGATCCACGGCGGTGGAATTAACACCCTTGACATTG	CSA C1	XhoI
CSA C2 Fw	ATTTTCTCGAGGCCACCATGGTTCACAGATACAGTGTGGAGACTGTACAGTGGTATC	CSA C2	
CSA C3 Fw	ATTTTCTCGAGGCCACCATGGGTCACAGACAAGAAATATTAGCAGTTTCCTGGTCTC	CSA C3	
CSA C4 Fw	ATTTTCTCGAGGCCACCATGGCTCATAATGGGAAAGTTAATGGCTTATGTTTTACAAGTG	CSA C4	
CSA C5 Fw	ATTTTCTCGAGGCCACCATGGGACATTATAAAACTGTTGACTGCTGTGTATT	CSA C5	
CSA Rv	ATTTTGGTACCGTTGCTCCTTCTTCATCACTGCTGCTCCAGGCATCTTCA	CSA C1-5	KpnI
CSA N1 Fw	CTAGCCGCCATGCCATCCTTATATGAACCAGTTCCTGATGATGATGAGACTACAACAAAATCACAA<br>TTAAATCCGGCCTTTGAAGATGCCTGGAGCAGCAGTGATGAAGAAGGAGCG	CSA N1	NheI/HindIII
CSA N1 Rv	AGCTTTCTTTCAACATCTCTGTCTTTATTTAATTCCAGTCCCAAAACTCTCCGTGTTGACTCTGCTCTC<br>CGAAGGCGAAGAGGGTCCTCCAAACCCGTTTGGCGTGCGGACAAAAACCCCAGCATGGCGG	CSA N1	
CSA C6 Fw	CTAGCCGCCATGCCATCCTTATATGAACCAGTTCCTGATGATGATGAGACTACAACAAAATCACAA<br>TTAAATCCGGCCTTTGAAGATGCCTGGAGCAGCAGTGATGAAGAAGGAGCG	CSA C6	NheI/HindIII
CSA C6 Rv	GATCCGCTCCTTCTTCATCACTGCTGCTCCAGGCATCTTCAAAGGCCGGATTTAATTGTGATTTTGT<br>TGTAGTCTCATCATCATCAGGAACTGGTTCATATAAGGATGGCATGGCGG	CSA C6	
CSB-285 Fw	AAAACTGTCTCTCGAGAGGAAGAAGCAAGGTTGTAATAAAAGAGCAGCTAG	CSB 285-1009	
CSB-302 Fw	AAAAGCTCCACTCGAGGTCACGCCTCCAGCCCCAGTGCAAAATAAAAAC	CSB 302-1009	

CSB-507 Fw	ATTTCTGTTCCTCGAGCTTTTTAAGTACCAGCAGACAGGTGTTAGGTGGCTGTGGGAA	CSB 507-1009,507-1192,507-1320,507-1493
CSB-1009 stop Rv	TCTGGGATCCTTATTAACTAGTCAG	CSB 285-1009,302-1009,507-1009
CSB-1192 stop Rv	TAAAAATGTTTCTCGGATCCTTATTATTCTGCCACACTATGATGTTTTGTTTTTGAC	CSB 507-1192
CSB-1320 stop Rv	TAAAAACCTGCGGATCCTTATTAAATCCCCCTGTGGCCAGTCCAGGTGGGAAC	CSB 507-1320
CSB-1493 stop Rv	TCTAGATCCGGTGGATCCTTATTAGCAGTATTCTGGCTTGAGTTTCCAAATTC	CSB 507-1493
NLS1 del Rv	TCAGTTTATTGCGGCCGCCTAACCGCTGCTTATAATAATCTTCATCTC	CSB ΔNLS1
NLS1 del Fw	ACAAAGAGAAACGCGGCCGCCTGGAGGACGATTCTGAGGAAAGTGATGCTG	CSB ΔNLS1
NLS2 del Rv	AAAGGCTGGTTGGCGGCCGCCTTTTAGATGGCATTTGGGTGTCTGAACATCT	CSB ΔNLS2
NLS2 del Fw	TTCCAAAACGCGGCCGCTTCCCTGCTTCTAACATATCTGTAAATGATGC	CSB ΔNLS2
NLS3 del Rv	ACAACCTTGGCGGCCGCCTTCAAAAGACAGTTTTGCTTGATCTGCCAAATACTT	CSB ΔNLS3
NLS3 del Fw	ATTTTTAAAGGCAAGCGGCCGCTGGGAGTCAGACATGAGGCCAGAGGCAGAGGGAG	CSB ΔNLS3
NoLS2 del Rv	ATGTTTCTCCAGGCGGCCGCCTTCTGCCACACTATGATGTTTTGTTTTTGACTTGTG	CSB ΔNoLS2
NoLS2 del Fw	AAACAAGAGTGGCGGCCGCGAACAGAGCAATGACGATTATGTTTTGGAAAAGC	CSB ΔNoLS2
NoLS3 del Rv	TTTCCTGCTGGGCGGCCGCCAATCCCCCTGTGGCCAGTCCAGGTGGGAAC	CSB ΔNoLS3
NoLS3 del Fw	TAACTTCTCTGGCGGCCGCCCTTCATCAACATCTCCAACAGAGAAGTGCCAGGAT	CSB ΔNoLS3
NoLS1 fragment Fw	AGCTTGTCACGCCTCCAGCCCCAGTGCAAAATAAAAACAAACCAAACAAGAAAGCCAGAGTTCTG TCCAAAAAAGAGGAGCGTTTGAAAAAGCACATCAAGAAACTCCAGAAGAGGGCTTTGCAGTAAT AAG	pGFP-fNoLS1
NoLS1 fragment Rv	GATCCTTATTACTGCAAAGCCCTCTTCTGGAGTTTCTTGATGTGCTTTTTCAAACGCTCCTCTTTTTT GGACAGAACTCTGGCTTTCTTGTTTGGTTTGTTTTTATTTTGCACTGGGGCTGGAGGCGTGACA	pGFP-fNoLS1
NoLS2 fragment Fw	TCGAGGAAGAGACCCTGGAGAAACATCTGAGACCAAAGCAAAAGCCTAAGAACTCTAAGCATTG CAGAGACGCCAAGTTTGAAGGAACTCGAATTCCACACCTGGTGAAGAAAAGGCGTTACCAGAAG CAAGACAGTGAAAACAAGAGTGAGGCCAAGG	pGFP-fNoLS2

NoLS2 fragment Rv	GATCCCTTGGCCTCACTCTTGTTTTCACTGTCTTGCTTCTGGTAACGCCTTTTCTTCACCAGGTGTGGAATTCGAGTTCCTTCAAACTTGGCGTCTCTGCAATGCTTAGAGTTCTTAGGCTTTTGCTTTGGTCTCAGATGTTTCTCCAGGGTCTCTTCC	pGFP-fNoLS2
NoLS3 fragment Fw	TCGAGTCTGGTGCACCAGCAGGAAAAAAGAGTAGATTTGGTAAGAAAAGGAATTCTAACTTCTCTGTGCAGCATG	pGFP-fNoLS3
NoLS3 fragment Rv	GATCCATGCTGCACAGAGAAGTTAGAATTCCTTTTCTTACCAAATCTACTCTTTTTTCCTGCTGGTGCACCAGAC	pGFP-fNoLS3
Ctrl NoLS Fw	TCGAGCCAGAAAGAAGCGAAAAAAGAAGAGAAAGAAGCGAAAAAAGAAGAGAAAGAAGCGAAAAAAGAAGG	pGFP-Ctrl NoLS
Ctrl NoLS Rv	GATCCCTTCTTTTTTCGCTTCTTTCTCTTCTTTTTTCGCTTCTTTCTCTTCTTTTTTCGCTTCTTTCTGGC	pGFP-Ctrl NoLS

Table S2. Primary antibody list. WB = western blot; IS = immunostaining.

Supplier	Antibody	Cat.No.	
Abcam	CSA	ab137033	WB
	CSB	ab66598	
	DDB1	ab124672	
	β-Actin	ab8224	
ABclonal	GAPDH	AC027	
Cell Signaling Technology	Histone H3	#4499	
	MEK1/2	#9122	
	Lamin A/C	#2032	
Santa Cruz Biotechnology	DDB2	sc-81246	
Sigma-Aldrich	β-Actin	A2228	
Thermo Fisher Scientific	Nucleolin (C23) clone 3G4B2	05-565	IS
	Nucleophosmin (B23)	32-5200	
	Alexa fluor 647 donkey anti-mouse IgG	A-31571	

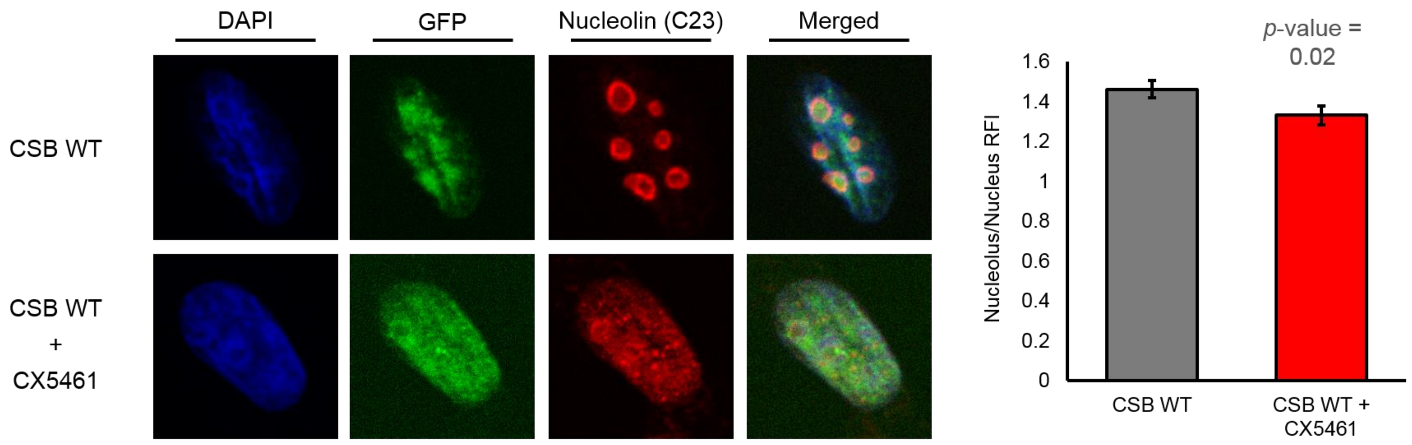

Figure S1. RNAPI inhibition reduces CSB nucleolar localization. HeLa cells were transfected with pCSB-GFP plasmid using the JetPrime reagent (Polyplus-transfection, Illkirch, France) and subsequently treated (or not) with 1 µM CX5461 for 24hr. CSB WT localization was determined by quantifying nucleolar (defined by nucleolin/C23 staining) and whole nucleus GFP intensity. Shown are representative images of DAPI, GFP and nucleolin (C23) for the different treatment groups captured by fluorescent microscopy (Zeiss Microscopy, Observer.Z.1, X-cite) (left), as well as the average and standard deviation of the nucleolar/nuclear RFI as determined by ImageJ software on 33 (control) and 21 (CX5461) independent cell images (right).

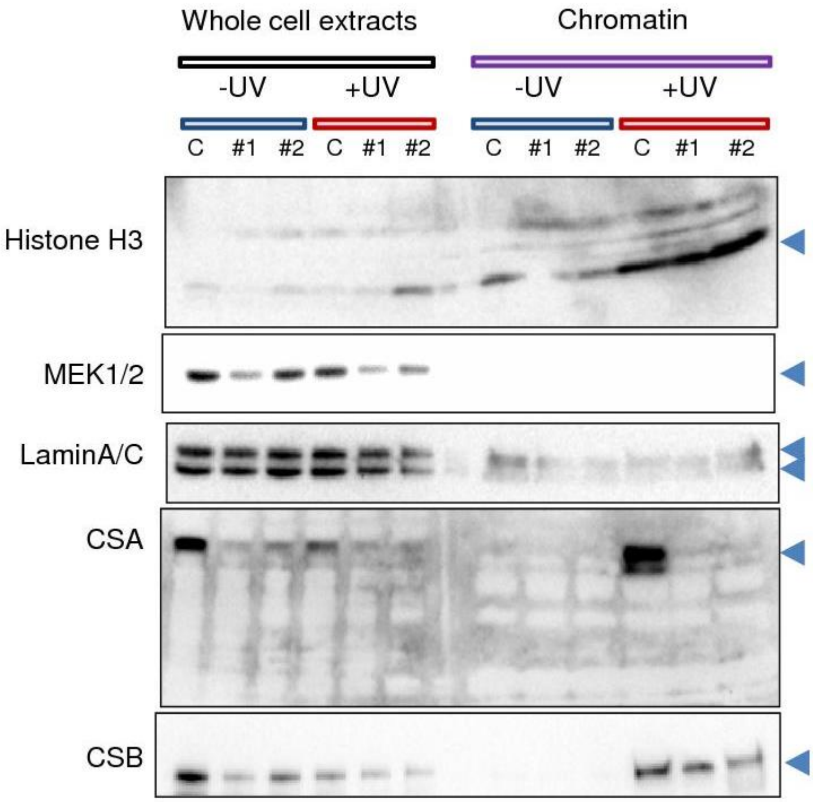

Figure S2. CSA chromatin association following UVC treatment is DDB1-dependent. U2OS cells were transfected with the control siRNA (C) or one of the DDB1-targeting siRNAs (#1 or #2), and subsequently UV irradiated (15 J/m^2) or not. Whole cell extracts and chromatin fractions were prepared 5 hr after irradiation, and then subjected to western blot analysis for CSA, CSB, histone H3 (chromatin marker), MEK1/2 (cytoplasm marker), or LaminA/C (nuclear marker).
